# TBP-like protein (TLP) interferes with Taspase1-mediated processing of TFIIA and represses TATA box gene expression

**DOI:** 10.1093/nar/gkv576

**Published:** 2015-06-01

**Authors:** Hidefumi Suzuki, Momoko Isogai, Ryo Maeda, Kiyoe Ura, Taka-aki Tamura

**Affiliations:** Department of Biology, Graduate School of Science, Chiba University, 1-33 Yayoicho, Inage-ku, Chiba 263-8522, Japan

## Abstract

TBP-TFIIA interaction is involved in the potentiation of TATA box-driven promoters. TFIIA activates transcription through stabilization of TATA box-bound TBP. The precursor of TFIIA is subjected to Taspase1-directed processing to generate α and β subunits. Although this processing has been assumed to be required for the promoter activation function of TFIIA, little is known about how the processing is regulated. In this study, we found that TBP-like protein (TLP), which has the highest affinity to TFIIA among known proteins, affects Taspase1-driven processing of TFIIA. TLP interfered with TFIIA processing *in vivo* and *in vitro*, and direct binding of TLP to TFIIA was essential for inhibition of the processing. We also showed that TATA box promoters are specifically potentiated by processed TFIIA. Processed TFIIA, but not unprocessed TFIIA, associated with the TATA box. In a TLP-knocked-down condition, not only the amounts of TATA box-bound TFIIA but also those of chromatin-bound TBP were significantly increased, resulting in the stimulation of TATA box-mediated gene expression. Consequently, we suggest that TLP works as a negative regulator of the TFIIA processing and represses TFIIA-governed and TATA-dependent gene expression through preventing TFIIA maturation.

## INTRODUCTION

The assembly of the transcription initiation complex on a promoter region is a critical step in gene expression. The TATA box is a promoter-proximal regulatory element that is responsible for high transcription efficiency of RNA polymerase II (polII)-dependent genes (1,2). It is known that over 20% of polII-driven promoters have a TATA box (3). Recruitment of general transcription factors (GTFs) determines the activity of TATA box-containing promoters (TATA promoters). TATA-binding protein (TBP), which is a main component of transcription factor IID (TFIID), recognizes and directly binds to the TATA box, and it recruits other GTFs to the promoter-proximal region (2,4–6). Transcription factor IIA (TFIIA) is another GTF and is known to be a binding partner of TBP (7–9). TFIIA enhances the transcription activity of TATA promoters through stabilization of binding of TBP to the TATA box. TFIIA directly binds to TBP and constitutes the core of the preinitiation complex with TFIIB. In addition, TFIIA has been reported to have an activator-like function (10,11), implying that transcription is regulated by TFIIA via various mechanisms. Otherwise, TFIIA is a biologically significant protein since it is essential for cell growth (12). However, compared with other GTFs, TFIIA has not been deeply investigated from mechanistic point of view in gene expression.

TFIIA consists of α, β and γ subunits (9). TFIIAα and β are encoded by single gene called TFIIA-L, which is referred to as TOA1 in budding yeast. The TFIIAαβ precursor is processed by Taspase1 at the QVDG site (13), and processed TFIIAα and β assemble into a holo-TFIIA complex together with TFIIAγ (α/β + γ). The holo-TFIIA complex has been thought to be a transcriptionally functional form of TFIIA. In the process of spermatogenesis, the TFIIAαβ precursor is processed by Taspase1 along with development, and holo-TFIIA potentiates the expression of spermatogenic genes (14). In cultured HeLa cells, most of the TFIIA proteins exist as processed forms. On the other hand, it has been reported that the unprocessed TFIIAαβ precursor itself also has a transcription activation function. An abnormality in the development of a TFIIA-deficient *Xenopus* embryo was rescued by the introduction of uncleavable TFIIA (13). It has also been reported that, unprocessed TFIIAαβ in P19 embryonic carcinoma cells forms a complex with TBP and TFIIAγ, which is referred to as TAC (TBP-TFIIA-containing) complex (15,16). Those reports suggest that processed and unprocessed TFIIAs each have a specific role in cell growth and development. The function of processed and unprocessed TFIIAs has therefore been studied in specific biological conditions like embryonic development. Moreover, the mechanistic investigation for each form of TFIIA has been performed just by *in vitro* analyses. Eventually, the intrinsic and general significance of the processing in transcriptional regulation is remained to be elucidated. Therefore, studies in functional differences between processed and unprocessed TFIIA and the regulatory mechanism of TFIIA processing are to be carried out by using commonly used cells such as HeLa cells.

TBP-like protein (TLP) was identified as one of the TFIIA-binding proteins with the highest affinity (17,18). TLP, also known as TRF2, is a member of the TBP-family proteins (19–22). Amino acids of TBP for binding to TFIIA and TFIIB are conserved in TLP, even though TLP does not bind stably to the TATA box sequence. It has been reported that the affinity of TLP to TFIIA is one order higher than that of TBP (18). We previously found that interaction between TLP and TFIIA is required for activation of TATA-less promoters (23–25). TLP and TFIIA regulate cell proliferation through activation of the upstream promoter of the *p21* gene in a p53-dependent manner. A recent study has also shown that TLP is engaged in potentiation of several types of TATA-less promoters of *Drosophila* (26,27). Furthermore, in an *in vitro* assay system, TLP inhibits TATA box-driven transcription by competing with TBP for TFIIA association (28). However, the *in vivo* role of TLP in TATA box promoters remains unclear.

In this study, we examined the effect of TLP-TFIIA interaction on TATA promoters and found that TLP represses TATA box-driven gene expression *in vivo*. We propose as a new mechanism that TLP represses promoter activity by preventing Taspase1-mediated processing of TFIIA. We also found that the TFIIAαβ itself is not involved in TATA box-mediated transcription activation and the processing of the TFIIAαβ precursor by Taspase1 is essential for full potentiation of TATA box promoters in cultured human cells. The role of TLP-TFIIA interaction is thought to be a critical determinant for expression of TATA box genes.

## MATERIALS AND METHODS

### Cell culture, transfection and drug treatment

Human HCT116 cells and HeLa cells were maintained in Dulbecco's modified MEM with high and low concentration of glucose, respectively (Sigma-Aldrich) at 37°C in the presence of 10% fetal calf serum. Knockdown experiments were performed as described previously (25). Lipofectamin2000 reagent (Invitrogen) was used for transfection of plasmids and siRNA. Cycloheximide (CHX) and MG132 dissolved in dimethyl sulfoxide (DMSO) were added to the media for some experiments.

### Plasmids

Mammalian expression plasmids: expression plasmids for flag/oligohistidine-tagged (FH)-TLP, FH-N37E, FH-TFIIA-L, FH-TFIIA-S and FH-MyoD were described previously (25,29). pCIneo-FH-TBP has an open reading frame of human TBP with an FH-tag at each of its amino termini. DGAA mutant of TFIIA-L was generated by a polymerase chain reaction (PCR)-based mutagenesis method from pCIneo-FH-TFIIA-L. Amino acids of the 274^th^ aspartic acid and 275^th^ glycine were substituted by alanine in the DGAA mutant.

Bacterial expression plasmids: open reading frames of *TBP, Taspase1, TFIIAαβ* and *TFIIAγ* were subcloned into a pET-3a vector (Novagen). An FH-tag was added to each of the amino terminal ends of the open reading frames of *TBP* and *Taspase1*, and oligohistidine (His)-tag was linked to that of *TFIIAγ*.

Reporter plasmids for luciferase assay: a pGL4.10 vector was used for construction of reporter plasmids. Promoter regions of human *p21* were amplified from human genomic DNA and cloned into a pGL4 vector by a standard PCR-based method. p21-168/GL4, p21-65/GL4 and p21-5/GL4 harbor distinct length of the p21 promoter as shown in Figure 2A(a). In the figure, +1 represents the transcription start site of a promoter gene. GAPDHWT/GL4, which carryies a promoter region from −145 to +52 of the human *GAPDH* gene, was also generated as described above. GAPDHmutTATA/GL4, which is a mutant construct of GAPDHWT/GL4, has a disrupted TATA box sequence.

**Figure 1.**
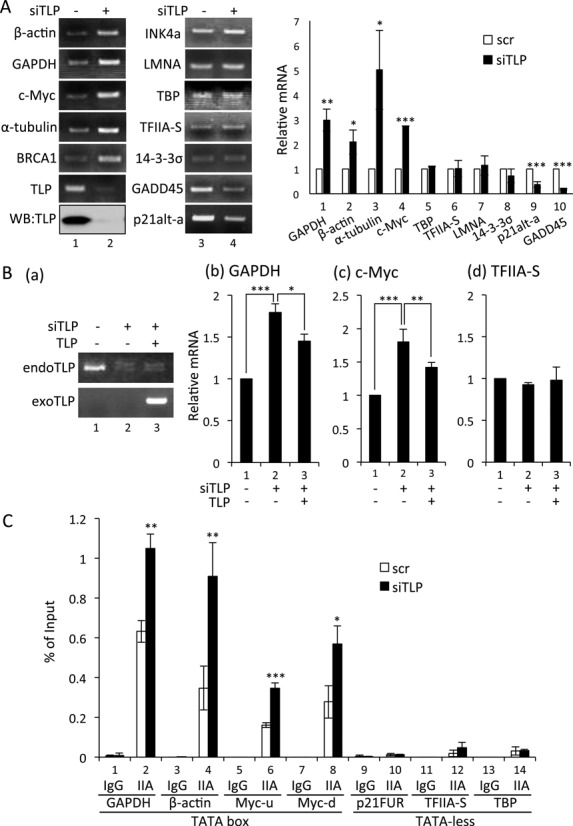
Negative function of TLP in endogenous gene expression. (**A**) Effect of TLP knockdown on endogenous gene expression of human cells. HeLa cells were transfected with TLP siRNA (siTLP) and control siRNA (scr), and amounts of mRNAs were determined by qRT-PCR. Relative amount of mRNA with siTLP (solid columns) to that with scr (open columns) for each of the genes is shown. Knockdown efficiency of TLP was checked by Western blotting (left panel). (**B**) Effect of exogenous expression of TLP on endogenous gene expression. HeLa cells were transfected with TLP siRNA together with a TLP expression plasmid. *TLP* mRNA was determined by semi-qRT-PCR (a). mRNAs of *GAPDH, c-Myc* and *TFIIA-S* were determined by qRT-PCR (b–d). (**C**) Amounts of chromatin-bound TFIIA. TLP-knocked-down HeLa cells were subjected to a ChIP assay using a TFIIAαβ-specific antibody. ChIP enrichment was determined by qPCR. IgG and IIA: IgG- and TFIIA-specific immnoprecipitates, respectively. Myc-u and Myc-d indicate upstream and downstream promoters of the *c-Myc* gene, respectively. p21FUR indicates the far-upstream region (-7018 to -6833) of the *p21* promoter used as a negative control.

**Figure 2.**
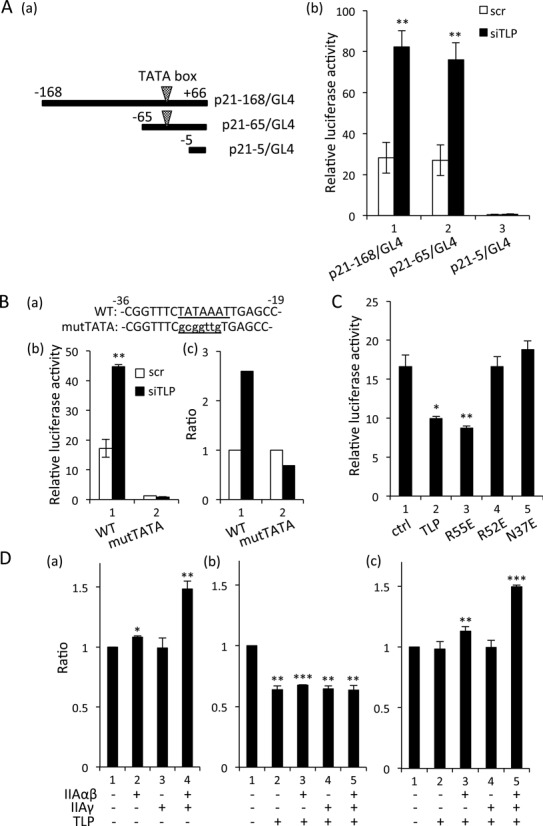
TLP-dependent repression of TATA promoters. (**A**) Luciferase reporter assay for TATA-containing human *p21* promoters. HeLa cells transfected with TLP siRNA together with the indicated *p21* promoter reporter were examined for relative luciferase activity. (a) Schematic representation of the promoter regions of the human *p21* gene inserted in p21-168/GL4, p21-65/GL4 and p21-5/GL4. Open arrowheads show the TATA box sequence. (b) Relative promoter activity was determined. (**B**) Luciferase reporter assay for TATA-containing human *GAPDH* promoter. Relative luciferase activity of the *GAPDH* promoter in TLP-knocked-down HeLa cells and the ratios were determined as described above. WT: wild-type TATA box-harboring *GAPDH* promoter. mutTATA: consensus TATA box sequence of the *GAPDH* promoter (TATAAAT) was mutated to gcggttg. (**C**) Effect of overexpressed TLP on *GAPDH* promoter activity. Wild-type TLP and its mutants R55E, R52E and N37E were introduced into HeLa cells together with a *GAPDH* promoter-containing reporter plasmid, and relative luciferase activity was determined. (**D**) Effect of co-expression of TLP and TFIIA on the *p21* promoter. HCT116 cells were transfected with TFIIA expression plasmids together with TLP (b) and N37E (c) expression plasmids, and the ratios of luciferase activity of p21-168/GL4 (vec = 1.0) are shown.

### PCR

Preparation of total cellular RNAs and reverse transcription PCR (RT-PCR) and quantitative PCR (qPCR) were performed as previously described (25,30). Semi-quantitative PCR was performed using Paq5000 DNA polymerase, and amplified products were analyzed by 2% agarose gel electrophoresis. All reactions were performed in triplicate. Primer sets to detect transcripts were as follows: β-actin forward, 5′-ACTGGGACGACATGGAGAAAA; reverse, 5′-GATAGCACAGCCTGGATAGCAA; GAPDH forward, 5′-GTCAAGGCTGAGAACGGGAA; reverse, 5′-AAATGAGCCCCAGCCTTCTC; c-Myc forward, 5′-CATCAGCACAACTACGCAGC; reverse, 5′-GCTGGTGCATTTTCGGTTGT; α-tubulin forward, 5′-CTGGCTTCACCTCACTCCTG; reverse, 5′-GAAGGCACAGTCTGAGTGCT; BRCA1 forward, 5′-GGTGGTACATGCACAGTTGC; reverse, 5′-ACTCTGGGGCTCTGTCTTCA; TLP forward, 5′-GGAAGATTGCTTTGGAAGGAGC; reverse, 5′-CCTGAGGACCAAATTGTAGCTG; INK4a forward, 5′-GAATAGTTACGGTCGGAGGC; reverse, 5′-GTACCGTGCGACATCGCGAT; LMNA forward, 5′-TCGCATCACCGAGTCTGAAG; reverse, 5′-ACTGAGTCAAGGGTCTTGCG; TBP forward, 5′-CTGGCCCATAGTGATCTTTGC; reverse, 5′-TCAATTCCTTGGGTTATCTTCACA; TFIIA-S forward, 5′-TTTGGGAAACAGTCTTCAGGA; reverse, 5′-CCATCACAGGCTACAATTTTCA; 14-3-3σ forward, 5′-AGAGCGAAACCTGCTCTCAG; reverse, 5′-CTCCTTGATGAGGTGGCTGT; GADD45 forward, 5′-ACGAGGACGACGACAGAGAT; reverse, 5′-GCAGGATCCTTCCATTGAGA; p21alt-a forward, 5′-CTGTTTTCAGGCGCCATGTC; reverse, 5′-GGTGGCTATTTTGTCCTTGG

### Luciferase reporter assay

A luciferase assay was performed as previously described (25). In a TLP-knocked-downed condition, siRNA for TLP was introduced into cells 36 h before transfection of reporter and effector plasmids. Cells were transfected with the indicated amounts of reporter plasmids and cultured for 12–24 h. Then the cells were disrupted with a Passive Lysis Buffer (Promega). Luciferase activity in lysates was determined by a Dual Luciferase Reporter Assay System (Promega). Amounts of transfected DNA were standardized with a control thymidine kinase promoter (Promega). In a single luciferase assay, luciferase activity was normalized with protein concentration of lysates determined by the BCA method.

### Immunoprecipitation

Cell extracts were prepared as previously described (18). Three hundred micrograms of the extract was used for immunoprecipitation. Extracts were mixed with a specific antibody and precipitated with protein G-Sepharose 4 Fast Flow (GE Healthcare Bioscience). Exogenous FH-tagged proteins in extracts were precipitated with anti-Flag M2 Affinity Gel (Sigma-Aldrich). Normal rabbit IgG (Santa Cruz) and IgG-Sepharose 6 Fast Flow (GE Healthcare Bioscience) were used as control antibodies. Bound proteins were eluted and detected by Western blotting as described before (18).

### Western blotting

Proteins were separated by SDS-PAGE, transferred to an Immobilon-P PVDF membrane (Millipore), and detected by an ImmnoStar Zeta (Wako) by using specific antibodies and appropriate horseradish peroxidase-conjugated secondary antibodies including anti (α)-rabbit IgG and α-mouse IgG. The primary antibodies used included α-p53 antibody (Santa Cruz), α-β-actin antibody (Sigma-Aldrich), α-GAPDH antibody (Santa Cruz), α-α-tubulin antibody (Santa Cruz), α-p21 antibody (Santa Cruz) and α-FLAG M5 antibody (Sigma-Aldrich). Anti-TLP, α-TFIIAαβ, α-TFIIAγ, α-TBP and α-TFIIB antibodies were used as antigen-purified antibodies.

### Chromatin immunoprecipitation (ChIP)

ChIP was performed by a previously described method (23). Briefly, cells were cross-linked with formaldehyde, followed by cell lysis and DNA-fragmentation by micrococcal nuclease. Endogenous proteins and exogenous FH-proteins were precipitated with a specific antibody using Protein G-Sepharose 4 Fast Flow (GE Healthcare Bioscience) and α-Flag M2 Affinity Gel (Sigma-Aldrich), respectively. Antigen-purified α-TFIIAαβ antibody and commercial α-TBP antibody (Santa Cruz) were used for ChIP analyses. Immnoprecipitated DNAs were purified and analyzed by qPCR. Primer sets for ChIP analysis were as follows: β-actin forward, 5′-TCCTCAATCTCGCTCTCGCT; reverse, 5′-GCCGCTGGGTTTTATAGGGC; GAPDH forward, 5′-CTCAAGACCTTGGGCTGGG; reverse, 5′-TCGAACAGGAGGAGCAGAGA; Myc-u forward, 5′-GGCGTGGGGGAAAAGAAAAA; reverse, 5′- CGTCCAGACCCTCGCATTAT; Myc-d forward, 5′-GAGGCTTGGCGGGAAAAAGA; reverse, CTCTGCCTCTCGCTGGAAT; IIA-S forward, 5′- CTTCCCTGACAAGGCTTGAGT; reverse, 5′- CAGAACTGAGCTGACGACCC; TBP forward, 5′-CTCAAGAGCTTCGCCCCTC; reverse, 5′-AATGTCACTTCCGCCAGTT.

### Electrophoretic mobility shift assay (EMSA)

EMSA was conducted with purified recombinant proteins. Double-stranded DNA carrying a GAPDH TATA box sequence (sense: CGGTTTCTATAAATTGAGCC) was labeled with ^32^P-γ-ATP as previously described (23), and 80 000 cpm of the DNA probe was used for each EMSA reaction. Fifty nanograms of TBP was mixed with 40 ng of TFIIAγ and 40 ng of processed or unprocessed TFIIAαβ in EMSA buffer (20 mM Hepes-KOH [pH 7.9], 5 mM MgCl_2_, 100 mM KCl, 0.2 mM EDTA, 5 mM dithiothreitol, 10% glycerol) and incubated at 37°C for 30 min to form TBP-TFIIA complex. Labeled probe DNA was then added to the mixture and incubated at 30°C for 45 min to form protein-DNA complex. Protein-DNA complexes were separated by 4% native PAGE and detected by autoradiography. If necessary, unlabeled probe DNA was added to the binding reaction as a competitor.

### Purification of bacterially expressed proteins

FH-TBP, FH-Taspase1, TFIIAαβ and His-TFIIAγ were transformed into BL21 (DE3) *E. coli*., and the recombinant proteins were induced by isopropyl-1-thio-β-D-galactoside. Cells were lysed with lysis buffer (500 mM NaCl, 10 mM imidazole, 1% Triton X-100, 10% glycerol, protease inhibitor mix [PI mix]). Proteins were purified from lysates using anti-Flag M2 Affinity Gel or Ni-NTA Agarose (QIAGEN). Although TFIIAαβ has no appending tags, it binds to Ni-NTA due to an oligohistidine moiety in the central region of the polypeptide. Affinity carriers were washed three times with lysis buffer, and M2 and Ni-NTA-bound proteins were eluted with FLAG peptide and imidazole, respectively.

For preparation of processed TFIIAα and β subunits, 1 μg of the TFIIAαβ precursor was mixed with M2 Agarose-bound FH-Taspase1 in a reaction buffer (20 mM Hepes-KOH [pH 7.9], 5 mM MgCl_2_, 100 mM KCl, 0.2 mM EDTA, 5 mM dithiothreitol, 10% glycerol) and incubated at 37°C for 1 h. M2 Agarose-bound FH-Taspase1 was eliminated from the TFIIA solution by centrifugation centrifuge. Processing of the TFIIAαβ precursor was checked by SDS-PAGE and silver staining.

### Cell fractionation

A method described by Xie *et al*. (31) was used for preparation of chromatin-free and chromatin-bound fractions. Cells were lysed with NETN100 buffer (100 mM NaCl, 1 mM EDTA, 0.5% NP-40, 20mM Tris-HCl [pH 7.5]), and the supernatant fraction was collected as the chromatin-free fraction. Chromatin pellets were washed twice with NETN100 buffer. The pellets were then suspended in NETN420 buffer (420 mM NaCl, 1 mM EDTA, 0.5% NP-40, 20mM Tris-HCl [pH 7.5]) and the supernatant material was collected as the chromatin-bound fraction.

### Statistical analysis

Data obtained in this study are shown as means ± standard error of means from at least three independent experiments. Statistical significance of quantitative data was determined by Bonferroni's method with R Console (ver.3.0.3). *P* < 0.05 was regarded as statistically significant. Statistical significance of differences between samples is shown in figures with asterisks such as *; *P* < 0.05, **; *P* < 0.01 and ***; *P* < 0.001.

## RESULTS

### Repression of TATA box genes by TLP

A subset of TATA-less promoters including *NF1, TAp63* and *p21* is potentiated by TLP through its recruitment to a promoter region (23–25,32,33). On the other hand, TLP is reported to have neither affinity to TATA box DNA nor a function for activation of TATA box-containing promoters (TATA promoters) (22). Indeed, chromatin-bound TLP was not detected in TATA promoters in our previous works. Rather, TLP was shown by *in vitro* analyses to repress TATA promoters (28,32). In this study, to elucidate the *in vivo* function of TLP in a TATA box, we first performed knockdown experiments to investigate effect of TLP on expression of endogenous genes. Knockdown of endogenous TLP increased mRNAs of TATA genes (*β-actin, GAPDH, c-Myc, α-tubulin* and *BRCA1*) (Figure 1A). In contrast, expression of TATA-less genes (*INK4a, LMNA, TBP, TFIIA-S, 14-3-3σ, GADD45* and *p21alt-a*) was not increased by TLP knockdown (Figure 1A). Rather, expression of *GADD45* and *p21alt-a* was decreased by TLP knockdown, suggesting that TLP potentiates a subset of TATA-less promoters. Additionally, expression of *GAPDH* and *c-Myc* was suppressed when TLP expression was recovered by introduction of a TLP expression plasmid (Figure 1B). These results demonstrate that TLP affects the expression of TATA genes.

We next examined the effect of TLP on the *in vivo* promoter-associating capacity of TFIIA. ChIP results confirmed that TFIIA was enriched in TATA promoters (Figure 1C). We found that TLP knockdown increased the amount of TATA box-bound TFIIA (Figure 1C). Considerable amounts of promoter-associating TFIIA were detected in TATA box-carrying *GAPDH, β-actin* and *c-Myc* promoters under a TLP-knocked-down condition (Figure 1C, lanes 1–8). The human *c-Myc* gene has two TATA promoters (34), and the amount of promoter-bound TFIIA was increased for both TATA elements (Figure 1C, lanes 5–8).

We further examined whether the TATA box sequence is critical for TLP-mediated repression of promoters. We performed a luciferase reporter assay using *p21* (*CDKN1A*) and *GAPDH* promoters. In a TLP-knocked-down condition, the TATA box-harboring *p21* promoter exhibited higher activity than that of the control one (Figure 2A). Similarly, the *GAPDH* promoter was significantly elevated by TLP knockdown, while mutation of a canonical TATA sequence to a non-TATA sequence abolished the TLP sensitivity (Figure 2B), indicating that TLP affects promoter activity via the TATA box sequence. Consistent with the results described above, TLP overexpression donwregulated the activities of TATA-containing *GAPDH* (Figure 2C) and *p21* promoters (data not shown). Additionally, we examined three TLP mutants R55E, R52E and N37E. While R55E has normal TFIIA binding ability, R52E and N37E have been shown to be defective just in TFIIA binding ability (18). TFIIA-reactive R55E exhibited a repression effect on TATA-promoters as the wild-type TLP did (Figure 2C, lanes 2 and 3). Notably, R52E and N37E, which do not interact with TFIIA, did not affect those promoter activities (Figure 2C, lanes 4 and 5), implying that TFIIA reactivity of TLP is required for repression of the TATA promoter by TLP. We then examined a synergistic effect of TLP and TFIIA on the TATA promoter. Exogenously expressed TFIIA potentiated the TATA promoter of *p21* (Figure 2D, panel a), and that promoter activation was suppressed by TLP overexpression (panel b), while the repression was not restored by N37E (panel c). These results suggested that, although endogenous TLP does not associate with the TATA promoter, it represses TATA-containing genes through inhibition of TFIIA activity needed for TATA promoters.

### Inhibition of TFIIA maturation by TLP

**Figure 3.**
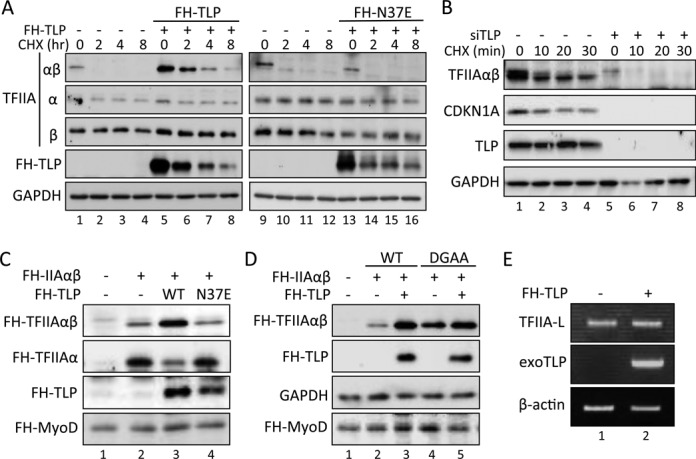
Inhibitory effect of TLP on the processing of TFIIAαβ. TFIIAαβ protein was determined by Western blotting. (**A**) Effect of overexpressed TLP on intracellular TFIIAαβ protein. TLP- and N37E-introduced HCT116 cells were treated with CHX for the indicated time, and the amount of endogenous TFIIA protein was determined. (**B**) Effect of TLP knockdown on TFIIAαβ precursor. The amount of endogenous TFIIAαβ precursor in TLP-knocked-down HeLa cells was assayed as described above. (**C**) Effect of TLP on TFIIAαβ processing. HeLa cells were transfected with expression plasmids for FH-TLP and FH-N37E together with FH-TFIIAαβ, and the TFIIAαβ precursor and generating α subunit were detected. (**D**) Effect of TLP on the uncleavable DGAA mutant. HeLa cells were transfected with expression plasmids of FH-TFIIA and FH-DGAA mutant together with TLP, and the amount of the FH-TFIIAαβ precursor was determined. FH-MyoD introduced into cells was used as an electrophoresis loading standard. (**E**) RT-PCR for detection of *TFIIA-L* transcripts. TLP-overexpressed HCT116 cells were assayed for *TFIIA-L* transcripts by RT-PCR using specific primers.

We next focused on the mechanism of TLP-mediated repression of TFIIA activity. Since the TFIIAαβ precursor is processed into mature α and β subunits by Taspase1 (13), the processing is a key step for intracellular TFIIA activity. To investigate the effects of TLP on TFIIA processing, we performed overexpression and knockdown experiments. The TFIIAαβ precursor has been reported to be rapidly processed into matured subunits in cells (35). Indeed, we found that the half-life of intracellular TFIIAαβ was less than 30 min. However, the amount of TFIIAαβ significantly increased in a TLP-overexpressed condition, and the half-life was prolonged to over 1 h (Figure 3A, lanes 5–8). Stabilization of the TFIIAαβ precursor was not observed for the N37E mutant (lanes 13–16). In turn, TLP knockdown resulted in destabilization of the TFIIAαβ precursor (Figure 3B). To demonstrate the inhibitory effect of TLP on nascent TFIIA protein, we examined the processing efficiency of TFIIAαβ by using exogenously expressed TFIIA. The exogenous TFIIAαβ precursor was processed into each subunit like the endogenous one (Figure 3C, lane 2). As expected, TLP, but not N37E, decreased the processing rate for exogenous TFIIAαβ (Figure 3C). Moreover, the protein level of DGAA mutant TFIIA, which is not processed by Taspase1, was not affected by TLP overexpression (Figure 3D). The amount of TFIIA transcript was not affected by TLP (Figure 3E). These results suggest that processing of TFIIAαβ precursor is inhibited by TLP.

**Figure 4.**
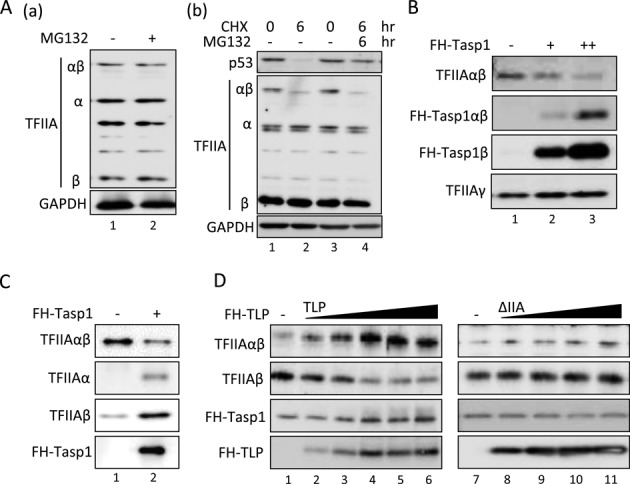
Inhibition of Taspae1-mediated processing of the TFIIAαβ precursor by TLP. (**A**) Effect of MG132 on TFIIA proteins. HCT116 cells were treated with MG132 and CHX for the indicated time, and the amount of TFIIA protein was determined. (**B**) Effect of overexpressed Taspase1 on the endogenous TFIIAαβ precursor. HCT116 cells were transfected with expression plasmids of FH-Taspase1, and the amount of the TFIIAαβ precursor was determined. (**C**) Taspase1-mediated processing of the TFIIAαβ precursor *in vitro*. Five nanograms of purified recombinant TFIIAαβ was mixed with 20 ng of purified recombinant FH-Taspase1 and incubated at 37°C for 1.5 h, and proteins were detected by Western blotting. Effect of TLP on Taspase1-mediated processing of the TFIIAαβ precursor *in vitro*. Twelve nanograms of TFIIAαβ and 6 ng of TFIIAγ were incubated with 50 to 200 ng of TLP or ΔIIA mutant protein at 37°C for 1 h. Fifty nanograms of FH-Taspase1 was then added to the mixture and incubated for 1.5 h.

We showed that the TFIIAαβ precursor was not degraded by the ubiquitin-proteasome system (Figure 4A). We further checked whether reduction of the precursor was due to Taspase1 (Figure 4B). We examined the inhibitory effect of TLP on *in vivo* processing of the TFIIAαβ precursor using purified recombinant proteins. Although the precursor of recombinant TFIIAαβ was sufficiently processed into α and β subunits by recombinant Taspase1 (Figure 4C), supplementation of TLP protein clearly prevented the processing of TFIIAαβ in a TLP-dose dependent manner, while the ΔIIA mutant of TLP, which has no affinity to TFIIA, did not affect the processing efficiency (Figure 4D). Taken together, the results showed that TLP inhibits Taspase1-driven processing of TFIIAαβ. Since TFIIA-binding ability-deficient TLP mutants did not affect the processing rate of the TFIIAαβ precursor, direct binding of TLP to TFIIAαβ was suggested to be critical for prevention of the processing.

### TATA promoter activation by processed TFIIAαβ

Since TLP repressed TATA-containing genes and inhibited processing of the TFIIAαβ precursor, we speculated that TLP negatively regulates TATA genes through inhibiting TFIIAαβ processing. However, it has remained ambiguous how the processing of the TFIIAαβ precursor functions in potentiation of TATA promoters.

**Figure 5.**
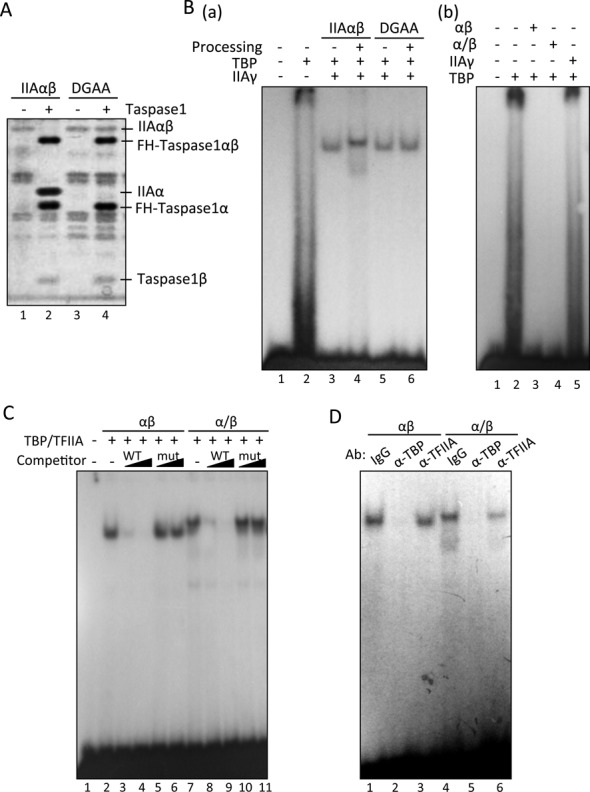
EMSA to detect the association of TFIIA with TATA the box. (**A**) *in vitro* processing of recombinant TFIIAαβ precursor protein. Twenty nanograms of purified recombinant TFIIAαβ and DGAA expressed in *E. coli* were mixed with 70 ng of purified recombinant FH-Taspase1 and incubated at 37°C for 1 h. Proteins were separated by SDS-PAGE and detected by silver staining. (**B**–**D**) EMSA of TFIIA and TBP to detect TATA box binding of the *GAPDH* promoter. Purified TFIIAαβ, His-TFIIAγ and FH-TBP were used. Panel B: (a) processed TFIIAαβ (indicated as Processing +, same with αβ of panel b) and unprocessed TFIIAαβ (indicated as Processing -, same with α/β of panel b) were used for EMSA. Processing: purified recombinant TFIIAαβ and DGAA were incubated with FH-Taspase1, and TFIIA proteins were purified. (b) Indicated combinations of purified proteins were used for EMSA. Panel C: cold probe DNA (WT) and its mutant (mut) were used as competitors in EMSA binding reactions. Sequences of wild-type and mutant competitors were 5′-CGGTTTCTATAAATTGAGCC and 5′-CGGTTTCCAGTAACTGAGCC, respectively. Panel D: specific antibodies against TBP, TFIIAαβ and control IgG were included in the EMSA. αβ and α/β indicate unprocessed and processed TFIIAαβ, respectively.

To clarify the significance of TFIIAαβ processing for promoter regulation, we performed an EMSA to examine the TATA box-association potential of processed and unprocessed forms of TFIIAαβ. First, we confirmed that purified recombinant TFIIAαβ was processed by Taspase1, whereas the DGAA mutant was not affected (Figure 5A). Although TBP alone did not stably bind to the TATA box, addition of TFIIA generated a higher protein (TBP-TFIIA)-DNA complex (Figure 5B). Unprocessed TFIIAαβ and processed TFIIAαβ generated two specific complexes. The combination of TBP, TFIIAγ and unprocessed TFIIAαβ or DGAA mutant generated a faster migrating complex (lower complex) (Figure 5B(a), lanes 3, 5 and 6). On the other hand, processed TFIIAαβ generated a slower migrating complex (upper complex) together with TBP and TFIIAγ (Figure 5B(a), lane 4). Since combinations of TBP and TFIIAαβ, or TBP and TFIIAγ did not generate stable protein-DNA complex (Figure 5B(b)), both TFIIAαβ and TFIIAγ were suggested to be required for formation of TBP-based protein-DNA complex. A competition assay revealed that these complexes were specific for the TATA box sequence (Figure 5C). We further performed a super-shift assay using specific antibodies to clarify the characteristic of these complexes. It was confirmed that both complexes contain TBP because these shifted bands reacted with a TBP-specific antibody (Figure 5D, lanes 2 and 5). However, these shifted bands exhibited different reaction to a TFIIA-specific antibody (Figure 5D, lanes 3 and 6). Because the lower complex did not react to TFIIA antibody, it was suggested that TFIIAαβ was not included in the lower complex, whereas processed form of TFIIAαβ was included in the upper complex. These EMSA results show that the processing step of TFIIAαβ is required for stable association of TFIIA with the TATA box.

**Figure 6.**
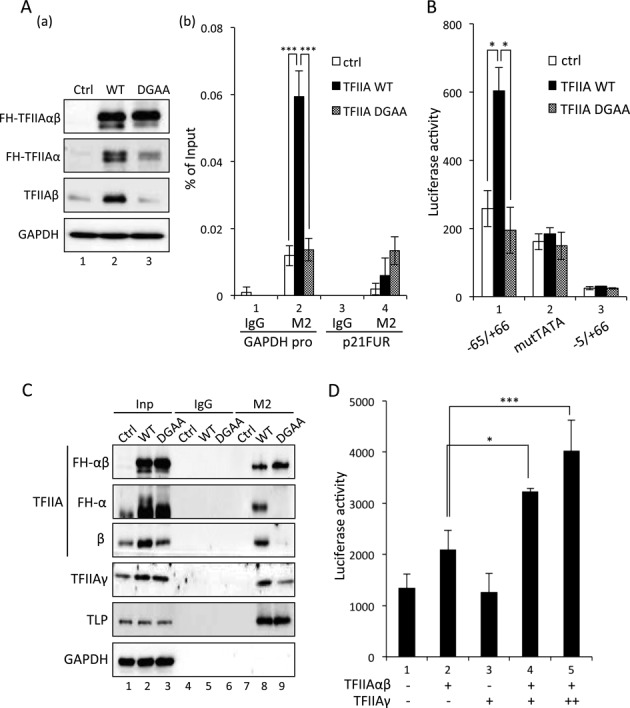
*In vivo* function of mature TFIIAα and TFIIAβ. (**A**) Chromatin binding of TFIIAαβ. Wild-type FH-TFIIAαβ (WT) and FH-DGAA (DGAA) were introduced into HeLa cells. Processing of wild-type TFIIAαβ was checked by Western blotting (a). Amounts of chromatin-bound exogenous FH-TFIIAαβ and FH-DGAA were determined by ChIP using M2 Agarose beads. ChIP enrichment at the TATA box-containing *GAPDH* promoter (GAPDH pro) and control DNA region (p21FUR) was determined by qPCR (b). Ctrl indicates control. (**B**) Activation of TATA promoter. Wild-type FH-TFIIAαβ and FH-DGAA were introduced into HeLa cells together with indicated *p21* reporter constructs, and the luciferase activity was determined. (**C**) Affinity of TFIIAαβ to its interacting proteins. Exogenously expressed FH-TFIIAαβ and FH-DGAA were immunoprecipitated with M2 Agarose beads, and co-precipitated TLP and TFIIAγ were detected. Inp: input. (**D**) A dose-responsive effect of TFIIAγ on TATA promoter activation. Indicated combinations of TFIIAs were introduced into HCT116 cells together with p21-65/GL4 reporter plasmid, and luciferase activity was determined. For dose-dependent analysis, 100 ng (+) or 200 ng (++) of TFIIAγ expression plasmid was used.

We performed ChIP analyses to determine whether the processing of TFIIAαβ is critical for its *in vivo* association with TATA promoters. Exogenously expressed TFIIAαβ was processed into the subunits (Figure 6A(a)). Processed TFIIA proteins were detected at the TATA promoter of the endogenous *GAPDH* gene, while the DGAA mutant was not detected at the promoter (Figure 6A(b)). Analysis of the promoter activation function of TFIIA showed that unprocessed TFIIAαβ is almost inert for the TATA-containing promoter (Figure 6B). To investigate the mechanism to achieve transcriptional activation by processed and unprocessed TFIIAαβ, we examined the interaction of the TFIIAαβ precursor (DGAA) with TFIIAγ. Although the TFIIAαβ precursor and processed TFIIA exhibited similar affinity to TLP, the TFIIAαβ precursor exhibited lower affinity to TFIIAγ (Figure 6C). The function assay revealed that TFIIAγ is required for TFIIAαβ-mediated potentiation of the TATA promoter. A dose-responsive effect of TFIIAγ on TFIIAαβ-mediated TATA promoter activation was observed (Figure 6D). The TFIIAγ subunit is thus thought to be required for potentiation of TATA promoters. Indeed, the EMSA showed that stable protein-DNA complexes were not generated in the absence of TFIIAγ (Figure 5B(b)). Consequently, it was clarified that processing of the TFIIAαβ precursor is required for its association with the TATA box and TFIIAγ-dependent potentiation of target promoters. Therefore, inhibition of TFIIAαβ processing by TLP is responsible for depression of TATA genes.

### Prevention of chromatin binding of TBP by TLP

**Figure 7.**
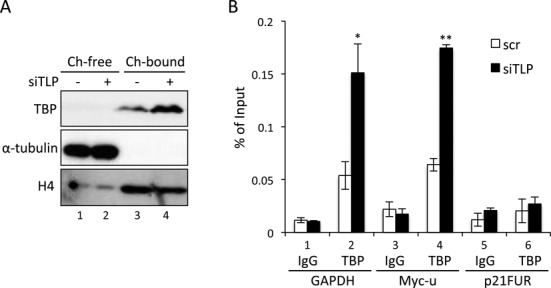
Negative effect of TLP on chromatin binding of TBP. (**A**) Amounts of chromatin-bound TBP. Chromatin-unbound (Ch-free) and chromatin-bound fractions (Ch-bound) were prepared from TLP-knocked-down HeLa cells, and the proteins were detected. Tubulin and histone H4 were used as standard proteins for chromatin-unbound and chromatin-bound fractions, respectively. (**B**) Amounts of promoter-bound TBP. TLP-knocked-down HeLa cells were subjected to a ChIP assay using a TBP-specific antibody. ChIP enrichment was determined by qPCR. IgG and TBP: IgG- and TBP-specific immnoprecipitates, respectively.

TBP is essential for potentiation of TATA promoters, and TFIIA supports TBP function through stabilization of the TBP-DNA association (9). Since TLP repressed TFIIA activity, it is speculated that TBP-DNA binding is affected by TLP. To confirm this hypothesis, we examined the amount of chromatin-bound TBP by cell fractionation and ChIP techniques. As expected, the amount of chromatin-bound TBP was markedly increased in a TLP-knocked-down condition (Figure 7A). Moreover, ChIP results showed that chromatin-bound TBP was specifically increased in the core promoter region (Figure 7B). This TLP-knockdown-dependent increase in TBP-chromatin binding was correlated with the increase in expression levels of TATA genes (Figure 1A). These results suggest that TLP works as a negative factor for TBP-driven transcription through inhibiting TFIIA function.

## DISCUSSION

### Repression of TATA genes by TLP

Since TLP is one of the TBP-family proteins, the role of TLP in transcription regulation has been studied (21,22). The most attractive characteristic of TLP is the highest affinity to TFIIA (17,18). In the transcription regulation of TATA-less promoters, several direct regulatory mechanisms of TLP have been shown over the past decade. We previously demonstrated that TATA-less promoters including *cyclinG2, TAp63* and *p21* need TLP-TFIIA interaction for transcription activation (23–25,33,36). Other groups demonstrated that several TATA-less promoters are also governed by TLP (e.g. TCT- and DPE-driven promoters and promoters of the histone gene cluster of *Drosophila*) (26,27). On the other hand, the mechanism by which TATA genes are affected by the TLP-TFIIA interaction *in vivo* has remained unclear. In this study, we showed that the TLP-TFIIA interaction has a negative effect on TATA genes (Figures 1 and 2). We found a novel mechanism that TLP inhibits TATA genes expression through preventing TFIIA maturation (Figures 3 and 4). Since 24% of the core promoters of human genes have a typical TATA box or TATA-like element (3), TLP may widely affect the expression of TATA genes as a global regulator.

### TLP behaves as an inhibitor of TFIIA processing

Sequence-specific cleavage of proteins is critical for regulation of cellular functions such as cell death, homeostasis and cell-cycle progression (37–39). TFIIAαβ has been demonstrated to be subjected to Taspase1-mediated proteolytic processing for production of mature TFIIAα and TFIIAβ (13). Although the processing of TFIIAαβ is known to be critical in spermatogenesis and head morphogenesis (14,40), the regulation mechanism of TFIIAαβ processing has remained unknown. We found that TLP functions as an inhibitor of TFIIAαβ processing (Figures 3 and 4). TLP has a specific and critical function for TFIIAαβ through its strong affinity to TFIIA. Results of this study suggest that direct binding of TLP to TFIIAαβ is required for inhibition of TFIIAαβ processing (Figures 3 and 4D). Although the amino acid sequence of TBP is 40% similar to that of TLP (19,28), TBP never inhibits the processing *in vivo* (data not shown). Our previous works have further shown that TLP has one-order higher affinity to TFIIA than TBP does (18). The high degree of stability of the TLP-TFIIA complex ensures the prevention of Tapsase1-mediated processing of the TFIIAαβ precursor. These observations suggest that TLP is not just a TBP alternative with inadequate ability but is a unique factor with a function distinct from that of TBP.

### TFIIAαβ processing is required for activation of the TATA promoter

Although the transcription activation function of TFIIA has been studied in yeast, yeast TFIIAαβ (TOA1) does not undergo proteolytic processing (13). On the other hand, the significance of Taspase1-mediated TFIIAαβ processing in human cells has not been elucidated. Although, from results of previous *in vitro* analyses, the Taspase1-mediated processing has been thought to be essential step for the TATA promoter activation (9), several studies demonstrated that the unprocessed TFIIAαβ has transcriptional activity (15,16,40). To demonstrate functional differences between each form of TFIIA clearly, we examined TFIIA function in conventionally used cultured cells. Finally, we showed that the processing of TFIIA is required for activation of TATA genes in human HeLa cells and HCT116 cells (Figure 6), and the unprocessed TFIIAαβ is inactive in transcription activation, which is explained below. We demonstrated that there are essential functional differences between processed and unprocessed forms of TFIIAαβ. Only processed TFIIAαβ is associated with the TATA box together with TBP and TFIIAγ (Figures 5 and 6). Moreover, processed TFIIAαβ has transcription activation function specifically for TATA promoters (Figure 6B). TFIIAγ is required for holo-TFIIA function (41), and the present study revealed that TFIIAγ is needed for both the TATA box associating ability and promoter activation function of processed TFIIAαβ (Figures 5B and 6D). In agreement with the promoter activation ability of processed TFIIAαβ, processed TFIIAαβ exhibited higher affinity to TFIIAγ than did its unprocessed form (Figure 6C). This considerable affinity is presumably needed for the TATA box-activation function of processed TFIIAαβ. Thus, the Taspase1-mediated processing of the TFIIAαβ precursor is required for acquiring both TATA box association ability and promoter activation function. Since processed TFIIAαβ can associate with the TATA box in the presence of TBP and TFIIAγ (Figure 5B), processed TFIIAαβ is likely to function as a co-activator of TBP together with TFIIAγ. Since the unprocessed TFIIAαβ does not exhibit such ability, this co-activator function is thought to be one of the mature TFIIAαβ-specific identities.

### Unprocessed TFIIAαβ can facilitate TBP-TATA box binding but is inactive in transcription activation

**Figure 8.**
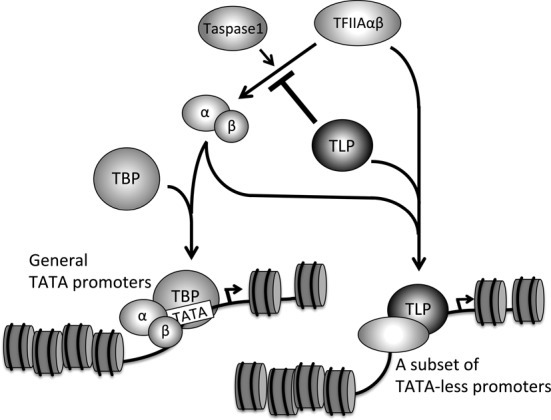
TLP-dependent promoter selection model of TFIIA. TLP inhibits Taspase1-mediated maturation of TFIIAαβ and represses TATA gene expression. TLP forms a complex with both the processed and unprocessed forms of TFIIAαβ and potentiates TLP-mediated genes. Consequently, TLP governs promoter selectivity of TFIIA and regulates gene expression.

TBP alone does not exhibit high affinity to the TATA box (3). This is probably because TBP is a sticky protein and tends to form functionally inactive homo-dimers (42,43). We previously found that TFIIA enhances the dissociation of TBP dimers (18,44). Unprocessed TFIIAαβ does not exhibit a stable TATA box-association function (Figures 5 and 6), but it facilitated the formation of a stable TBP-TATA box complex in solution (Figure 5). Other groups also reported that TBP-TATA box binding is stimulated by unprocessed TFIIAαβ (8,41). Hence, even the unprocessed TFIIAαβ can dissociate TBP dimers and orientate a TBP monomer to the TATA box. Because processed TFIIAαβ also facilitated TBP-TATA box association (Figure 5), the TBP-TATA stabilizing function of TFIIAαβ is restored after processing. So far, TATA box-association of TFIIA has been assumed to be required for orientation of TBP to a TATA box. Data obtained in this study, however, suggest that even the TFIIAαβ precursor, which does not exhibit association ability with the TATA box, basically has TBP-TATA box stabilization ability, and Taspase1-directed processing enables TFIIA to associate with the TATA box for full potentiation of TATA promoters as mentioned above. Consequently, we hypothesize that there are two steps for TFIIA-mediated potentiation of TATA promoters. The first step is orientation of a TBP monomer to TATA box DNA without association of TFIIA with the TATA box. The second step is TATA box-binding of TFIIA itself for activation of an associating promoter. Moore *et al*. demonstrated by *in vitro* analysis that TLP prevents TBP-TATA box complex formation as the first step through competing with TBP for TFIIA binding (28). We clearly showed that TLP prevents TBP-TATA box binding *in vivo* (Figure 7), probably due to disruption of TBP-TFIIA interaction by TLP. On the other hand, it might be possible that TLP disrupts TBP-TATA box binding via other mechanisms such as alternation of chromatin structure (45). In any cases, the present study demonstrated clearly that the Taspase1-mediated processing is a novel step for TLP to inhibit TATA-containing genes (Figure 8).

### TLP participates in promoter selectivity of TFIIA

We demonstrated that unprocessed TFIIAαβ is inactive in TATA promoter activation. On the other hand, unprocessed TFIIAαβ has much more capacity to activate a subset of TLP-dependent TATA-less promoters (Figure 8). We found that unprocessed TFIIAαβ activates the upstream promoter of the human *p21Cip1* gene (our unpublished data), which is rather dependent on TLP. Actually, promoter recruitment of unprocessed TFIIAαβ has been reported. Takeda *et al*. demonstrated that unprocessed TFIIAαβ associates with TATA-less promoters of *p16Ink4a* and *p19Arf* genes and enhances expression of those genes during mouse craniofacial morphogenesis (40). Thus, the TFIIAαβ precursor is likely to activate TBP-independent and TLP-governed TATA-less promoters. Because TLP also interacts with TFIIB, another GTF, TLP-governed promoters can be regulated by a specific transcription preinitiation complex containing TLP, unprocessed TFIIA and TFIIB.

We propose a TLP-governed promoter selection model of TFIIA (Figure 8). TLP inhibits processing of the TFIIAαβ precursor and accumulates the precursor. Although inhibition of TFIIAαβ maturation by TLP results in repression of TATA genes, TLP and accumulating unprocessed TFIIAαβ are cooperatively recruited to TATA-less promoters and potentiate a subset of genes.
